# Effect of Dietary Supplementation with Rumen-Protected GABA (γ-Aminobutyric Acid) on Milk Productivity and Blood Profiles of Dairy Cattle Under Heat Stress Conditions

**DOI:** 10.3390/ani16020262

**Published:** 2026-01-15

**Authors:** Young Hye Joo, Jun Sik Woo, Honggu Lee, Won Seob Kim, Keun Kyu Park, Yognjun Choi

**Affiliations:** 1 Department of Marketing, Milae Bioresources Co., Ltd., Seoul 05836, Republic of Korea; legend418@hanmail.net; 2 Central Research Institute, Harim Co., Ltd., Daejeon 08793, Republic of Korea; 3 Department of Animal Science and Technology, Konkuk University, Seoul 05029, Republic of Korea; 4 Department of Animal Resources, Daegu University, Daegu 38453, Republic of Korea; 5 School of Animal Life Convergence Science, HanKeung National University, Anseong 17579, Republic of Korea

**Keywords:** rumen-protected γ-aminobutyric acid, milk productivity, blood profiles, heat stress, lactating dairy cow

## Abstract

This study was conducted to evaluate the effect of rumen-protected γ-aminobutyric acid (GABA) supplementation on milk productivity of lactating Holstein cows. Eighteen Holstein dairy cows (mean parity, 2.2 ± 1.0 year; mean milk yield, 34.3 ± 5.5 kg) were selected in a commercial dairy farm for the experiment. The experiment was conducted from 17 July 2024 to 11 September 2024 (56 days). Generally, THI 72 is set as a threshold since the productivity of Holstein cows starts to decrease. Animals were exposed to heat stress conditions (THI ≥ 72) during the experimental period. The basal diet was fed as a total mixed ration (TMR), and GABA was top-dressed onto the TMR. The treatments were basal diet (Control), basal diet supplemented with rumen-protected GABA 3 g/d (Treatment 1), and basal diet supplemented with rumen-protected GABA 6 g/d (Treatment 2) as a completely randomized design. Statistical significance was compared between the control and GABA treatment groups using the method of repeated measurement. Increased levels of rumen-protected GABA supplementation tended to mitigate the decline in milk yield associated with heat stress (*p* = 0.083). Milk fat content in the GABA supplementation groups was significantly greater than that in the control group (*p* = 0.036). Milk lactose content was significantly increased by GABA supplementation (*p* = 0.017). Blood metabolic profiles and cortisol did not differ significantly between the control and GABA supplementation groups. Activities in the GABA supplementation groups were significantly greater than those in the control group (*p* < 0.05). Rest and rumination times in the GABA supplementation group were significantly lower than those in the control group (*p* < 0.05). These results suggest that rumen-protected GABA can be a practical nutritional intervention for minimizing productivity losses in Holstein cows during periods of elevated ambient temperature.

## Simple Summary

This study evaluated the effects of rumen-protected γ-aminobutyric acid (GABA) supplementation on milk productivity, blood metabolites, and behavior in heat-stressed Holstein cows. Eighteen cows were assigned to three groups—control, 3 g/d GABA, and 6 g/d GABA—and fed a basal TMR diet over 56 days in summer. Though overall milk production did not differ significantly, higher GABA supplementation tended to lessen heat-related yield declines. At week 8, both GABA groups showed ~4% higher yields versus baseline, while the control did not improve. Significant increases in milk fat (*p* = 0.036) and lactose (*p* = 0.017) were observed in GABA-supplemented cows. These findings suggest that rumen-protected GABA helps mitigate heat stress impacts and supports milk yield and composition in Holstein cows during hot periods.

## 1. Introduction

Since industrialization, global warming has been occurring worldwide, including in Korea, with unprecedented climate change manifesting particularly since the latter half of the 20th century [1]. Examining changes in seasonal duration, summer periods with average temperatures of 25 °C or higher lasted 98 days during an earlier 30-year period (1912–1941), whereas in a recent 30-year period (1988–2017), this figure increased to 117 days, representing a 19-day extension in exposure to high summer temperatures [2]. These climate changes directly impact the agricultural sector and represent a major factor causing decreased livestock productivity and economic losses in the livestock industry.

In response to heat stress, animals activate both physiological and behavioral heat dissipation mechanisms. Dermal arteriole dilation redirects blood flow to the skin surface to promote heat exchange with the environment, while increased sweating and mouth breathing enhance evaporative cooling [3,4]. Concurrently, behavioral adjustments including shade-seeking and reduced activity help minimize metabolic heat generation and support thermoregulation [5]. Heat stress induces various physiological changes in dairy cows, affecting their behavior, metabolism, and productivity [6]. When the temperature–humidity index (THI) exceeds 72, dairy cows begin to experience moderate heat stress [7], and break points which seriously decrease productivity are reported around THI values of 80–82 for milk yield of Holstein cows in South Korea [8]. Climate changes directly lead to decreased milk yield and cause multiple productivity losses, including alterations in milk composition, reduced reproductive efficiency, and increased disease incidence, which ultimately have direct negative impacts on farm profitability.

The rumen serves as a fermentation vat where microbial metabolism generates considerable metabolic heat. During heat stress, ruminants reduce feed intake to minimize the thermogenic effect of digestion and lower overall body heat production [9]. This decrease in feed intake is one of the primary factors contributing to the decline in milk production in dairy cows. Rumen temperature typically exceeds rectal temperature under normal feeding conditions due to heat generated by microbial fermentation [10]. Therefore, feeding nutrients with rumen-undegradable characteristics can sustain nutrient intake while minimizing increases in core body temperature.

Nutritionally, GABA is catabolized by mitochondrial GABA transaminase (GABA-T) to form succinic semialdehyde, which is subsequently oxidized to succinate and incorporated into the tricarboxylic acid (TCA) cycle, thereby contributing to cellular energy production [11]. In high-temperature environments (THI 78–80 or higher), cows supplemented with GABA exhibit greater feed intake as well as increased milk protein and lactose content compared to controls [12,13]. Supplementing 0.8–1.2 g/day of GABA has been shown to increase milk production and milk protein yield, reduce non-esterified fatty acids (NEFAs; indicating improved energy metabolism), and raise antioxidant markers such as glutathione peroxidase and superoxide dismutase [14]. Consequently, the provision of ruminally protected GABA may mitigate heat stress-induced decrements in productive performance, despite the paucity of research addressing this hypothesis. Therefore, this study was conducted to evaluate the effect of rumen-protected GABA supplementation on milk productivity of lactating Holstein cows.

## 2. Materials and Methods

### 2.1. Animal Care

All procedures were approved by the Animal Experiment Ethics Committee of Konkuk University (approval no. KU24153). Eighteen Holstein dairy cows (BW, 285.6 ± 45.9; mean parity, 2.2 ± 1.0 year; mean milk yield, 34.3 ± 5.5 kg) were selected in a commercial dairy farm (latitude, 37.205330; longitude, 127.653548) for the experiment (Table 1). Dairy cow characteristics are presented in Table 1 by experimental treatment. Feed was offered twice daily at 07:00 and 17:00 h, and water and mineral blocks were fed ad libitum.

### 2.2. Feeding Trials

The experiment was conducted from 17 July 2024 to 11 September 2024 (56 days). The basal diet was fed as a total mixed ration (TMR, Table 2), and each cow was restricted to 21.26 kg DM/day to maintain consistent feed intake across treatments. Each animal was housed in an individual pen for feeding, and residuals were not observed during experimental periods. Treatments were basal diet (Control), basal diet supplemented with rumen-protected GABA 3 g/d (Treatment 1), and basal diet supplemented with rumen-protected GABA 6 g/d (Treatment 2). Rumen-protected GABA was top-dressed onto the basal diet. The GABA supplement was prepared by coating GABA with calcium-salt-protected fat and hydrogenated oil, to achieve a final GABA concentration of 35% of dry matter. The basal diet was formulated based on the nutrient requirements of dairy cattle (NRC, 2001) [7]. Milking was performed twice a day at 03:00 and 15:00 h, and the daily milk yield was recorded.

### 2.3. Temperature–Humidity Index

THI was calculated from temperature and humidity data recorded hourly using an automatic thermo-hygrometer (MHT-381SD, Lutron Electronics Inc., Coopersburg, PA, USA) during the experimental periods. The THI affecting dairy cows was calculated using the THI prediction model presented by NRC [7]. The THI calculation formula is as follows:

THI = (1.8 × T_db_ + 32) − [(0.55 − 0.0055 × RH) × (1.8 × T_db_ − 26)] (1)

The THI ratio indicated in this study was calculated using the method of Nam et al. [15]. The THI ratio calculation formula is as follows:

THI ratio = 1 × Daily percentage under THI 72 − 1 × Daily percentage under THI 72 to 78 − 2 × Daily percentage under THI 78 to 89 − 3 × Daily percentage over THI 90.(2)

### 2.4. Milk Composition Analysis

For milk component analysis, samples were collected weekly using a sampler attached to a tandem mechanical milking machine (Matatron 21, GEA Westfalia Surge, Bönen, Germany). Sampling occurred twice on each sampling day, in the morning and afternoon; 50 mL of milk was collected, preserved with Broad Spectrum Microtabs II (ADVANCED Instruments, Inc., Norwood, MA, USA), and stored at 4 °C until analysis. Milk components were analyzed from combined samples of morning and afternoon milk. Milk fat, milk protein, solids-not-fat, milk urea nitrogen (MUN), and somatic cell count were measured using an automatic milk component analyzer (Milko-scan FT 6000, Foss Electric Co., Hillerød, Denmark). The 3.5% fat-corrected milk (3.5% FCM), fat- and protein-corrected milk (FPCM), and energy-corrected milk (ECM) were calculated following the formulas suggested by the method of NRC (2001) [7].

### 2.5. Blood Profiles

Blood samples were collected before feeding at 07:00 h on weeks 0, 4, and 8 of the experimental periods. Blood samples were collected from the jugular vein using a 20 mL syringe for analyzing common blood cells (CBCs) and metabolic profiles. Total blood was collected using a 10 mL EDTA tube (BD-367861, BD Vacutainer, Plymouth, UK) stored at 4 °C until CBC analysis. The CBCs were analyzed using a veterinary hematology analyzer (BC-2800Vet, Mindray, Shenzhen, China). Serum was separated into 10 mL serum blood collection tubes (BD-367820, BD Vacutainer, Plymouth, UK) and stored at 4 °C until the metabolic profile test (MPT) and cortisol analysis. The MPT includes total protein, glucose, total cholesterol, and non-esterified fatty acid. The MPT parameters and cortisol were analyzed using an automatic chemical analyzer (Cobas E801, Roche Holding AG, Basel, Switzerland).

### 2.6. Rectal Temperature and Behaviors

Rectal temperature was measured by inserting a thermometer (TC-300; TOOLCON, Bucheon, Republic of Korea) at least 5 cm into the rectum. Behavioral changes were monitored using an electronic ear tag (Cow Manager ear tag, Cow Manager BV, Hamelen, The Netherlands) attached to the animals’ ears, which recorded behavior continuously for 24 h. Five activity states were identified: high activity, activity, rest, feeding, and rumination (Table 3).

### 2.7. Statistical Analysis

Data were analyzed using a MIXED procedure of the SAS 9.4 software (SAS Inst. Inc., Cary, NC, USA) [16] as a completely randomized design. The model was

Y_ijk_ = μ + G_i_ + W_j_ + GW_k_ + E_ikj_,

where μ is the average value, G_i_ is the GABA supplementation effect, W_j_ is the time effect, GW_k_ is the interaction between GABA and the time effect, and E_ij_ is the error value. The fixed effect was GABA, and random effect was not considered. The interaction between GABA supplementation and sampling time was assessed to isolate GABA-specific effects. Statistical significance was compared between the control and GABA treatment groups using the method of repeated measurement [17]. Least square means between treatments were assessed using a pairwise comparison method. Statistical difference and tendency were accepted at a *p*-value less than 0.05 and 0.05 < *p* ≤ 0.10, respectively. All means are presented as least square means.

## 3. Results

### 3.1. Ambient Temperature, Relative Humidity, and Temperature–Humidity Index

The ambient temperature, relative humidity, and THI during the entire experimental period are presented in Table 4.

During the experimental period, the ambient temperature (AT) ranged from 26.7 °C to 29.5 °C, and relative humidity (RH) fluctuated between 79.2% and 99.9%, reflecting persistently hot and humid summer conditions. The THI values remained elevated throughout, with weekly mean THI spanning from 75.7 to 82.9, and weekly maximum THI consistently exceeding 80.

THI classification revealed that for the majority of the experiment (weeks 0 to 6), minimal to no time was spent at THI below 72, indicating that cows were constantly exposed to at least moderate heat stress. Instead, most time was spent in THI ranges of 73–80 and 81–89, with the latter representing severe heat stress. Only in the final weeks (weeks 7 and 8) did the proportion of THI values below 72 increase, signaling some relief from heat stress conditions.

The calculated THI ratio further confirmed the predominance of high heat stress, with negative values ranging from −71.0 to −177.4, and the most extreme negative ratios occurring during weeks 2 to 5.

### 3.2. Milk Yield and Composition

The effects of dietary GABA supplementation on milk yield and rectal temperature (RT) are presented in Table 5. Milk yield in the GABA supplementation group tended to be higher than that in the control group (*p* = 0.083), and 3.5% fat-corrected milk (FCM) in the GABA supplementation group tended to be higher than that in the control group (*p* = 0.088). Both 3.5% FCM and fat- and protein-corrected milk (FPCM) differed significantly among experimental weeks (*p* < 0.001). Rectal temperature did not differ between treatments (*p* > 0.05). No significant interactions between GABA treatment and week were observed for any parameter (*p* > 0.05).

**Figure 1:**
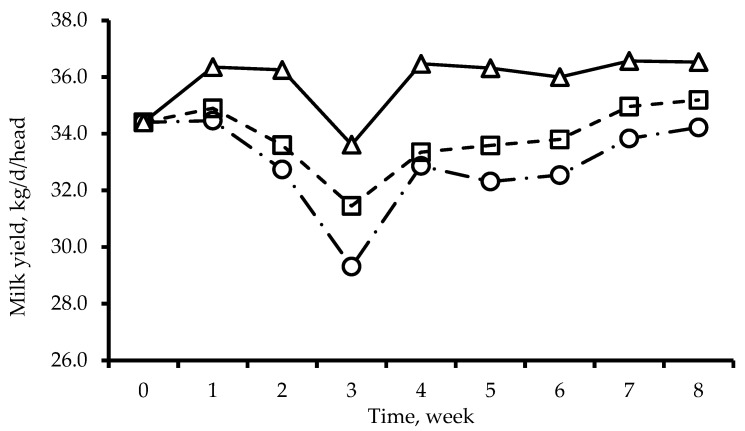
Milk yield during experimental period. Control (○), no additives; T-1 (□), rumen-protected GABA 3 g/head/day; T-2 (△), rumen-protected GABA 6 g/head/day.

The effect of dietary GABA supplementation on milk yield across experimental periods is presented in Figure 1. Milk yield in all treatments decreased from week 1 to week 3, followed by recovery after week 3 (Figure 1). Milk yield was lowest at week 3 in all treatments. Overall, milk yield in the GABA supplementation group was higher than that in the control group throughout the experimental period (Figure 1).

### 3.3. Milk Composition

The effects of dietary GABA supplementation on milk composition are presented in Table 6. Milk fat content in the GABA supplementation groups was significantly greater than that in the control group (*p* = 0.036). Milk protein content in the GABA 6 g treatment group tended to be lower than that in the control and GABA 3 g treatment groups (*p* = 0.055). Milk lactose content was significantly increased by GABA supplementation (*p* = 0.017). Milk solids-not-fat, milk urea nitrogen (MUN), and somatic cell count did not differ significantly among treatments. Week had a significant effect on all milk composition parameters; however, no significant interactions between GABA treatment and week were observed.

### 3.4. Metabolic Profiles and Cortisol

The effects of dietary GABA supplementation on metabolic profile test (MPT) parameters and cortisol are presented in Table 7. Glucose, albumin, total cholesterol, non-esterified fatty acid (NEFA), blood urea nitrogen (BUN), total protein (TP), aspartate aminotransferase (AST), alanine aminotransferase (ALT), Ca, p, and Mg did not differ significantly between the control and GABA supplementation groups. Cortisol concentration did not differ significantly between the control and GABA supplementation groups.

### 3.5. Behaviors

The behavior patterns of Holstein cows supplemented with dietary GABA throughout the experimental period are presented in Table 8. High activity and total activity in the GABA supplementation groups were significantly greater than those in the control group (*p* = 0.001 and *p* = 0.017, respectively). Resting behavior in the control group was significantly greater than that in the GABA supplementation groups (*p* = 0.043). Eating time did not differ significantly among treatments; however, it tended to increase with GABA supplementation (*p* = 0.051). Rumination time in the control group was significantly greater than that in the GABA supplementation groups (*p* = 0.001). No significant interactions between GABA treatment and week were observed for any behavioral parameter.

## 4. Discussion

Heat stress of livestock animals is mainly evaluated using THI, which is calculated using temperature and relative humidity [18]. The THI is classified by the productivity of the livestock animal, and it has four fractions. Generally, a THI threshold of 72 is used, as Holstein cow productivity begins to decline at or above this value [19]. The THI classification was divided into normal (THI under 68), mild (THI 68 to 72), moderate (THI 72 to 80), severe (THI 80 to 90), and danger zone (THI over 90). A previous study used a slightly different scale (e.g., normal < 70, alert 70–78, danger 79–82, emergency > 82), but similarly identified THI values above 72 as indicative of heat stress onset [20]. In this study, during week 0 to week 6, minimum THI was shown to be greater than standard thresholds (THI 72) of other studies and meant that most experimental cows were affected by heat stress during experimental periods. As maximum THI was shown to be under 90 during the entire experimental period, it is considered that it did not affect mortality risks. The decline in milk yield began after week 1, when the daily proportion of time under severe heat stress conditions (THI 80 to 89) exceeded the proportion of time below the moderate heat stress between weeks 1 and 2 (Table 3 and Figure 1). Recovery of milk yield was observed from week 4 onward (Figure 1), corresponding to a decrease in daily exposure to severe heat stress conditions (THI 80–89) between weeks 3 and 4 (Table 3). Following the return of THI values below the heat stress threshold after week 6, rapid recovery of milk yield was observed in all treatments, with substantial improvement evident within 7 days (between weeks 6 and 7). Previous research has demonstrated that milk yield can decrease by 35% during 10 days of severe heat stress exposure (THI 84), with 60% recovery of the lost production achieved within 14 days post-stress [21]. In this study, milk yield of the control group declined by a maximum of 14.7% during the period of increasing severe heat stress exposure, reaching the lowest point in week 3. Following the reduction in severe heat stress, milk yield recovered by approximately 8.8% and remained at this level through week 6. However, in a previous study, it was reported that the THI ratio threshold for a decline in productivity was −100 according to the criteria calculated by the method of Nam et al. [15], while in the present study, a decrease was observed starting at −144.4. This may indicate that the constant interval values used for THI ratio calculation [15] do not universally ensure accuracy across all studies. To improve model performance, it would be advisable to collect temperature, humidity, and flow rate data under a variety of environmental conditions and refine the model accordingly.

In this study, all animals received the same amount of feed under restricted feeding conditions and were exposed to identical heat stress environments. Therefore, GABA supplementation was the only difference between treatment groups. Nutritionally, GABA is metabolized by mitochondrial GABA transaminase (GABA-T), producing succinic semialdehyde, which is then converted to succinate and enters the tricarboxylic acid (TCA) cycle, thereby linking GABA metabolism to cellular energy production [11]. Specifically, GABA, which escapes rumen degradation, can directly participate in carbohydrate metabolism without conversion to volatile fatty acids. This may explain the increased milk yield observed when rumen-protected GABA was supplemented. Feed intake is a major source of heat generation in ruminants, and reducing feed intake serves to lower metabolic heat load during heat stress conditions [16]. Although ruminal temperature was not measured in this study and GABA did not affect rectal temperature, previous research demonstrated a strong correlation between daily THI and rumen temperature during heat stress [15], suggesting that heat stress in animals is closely associated with external environmental conditions. Rumen-protected GABA, which bypasses degradation in the rumen, can be directly utilized as a nutrient by the animal. This approach is believed to help reduce body heat gain and provide an effective source of energy, particularly under heat stress conditions.

In particular, in this study, the milk yield in the GABA treatment group was consistently higher than that of the control group during the entire experimental period (Figure 1), and the trends observed in both milk yield and 3.5% fat-corrected milk (FCM) indicate the positive effect of GABA supplementation (Table 5). Direct correlations between GABA and milk fat are less frequently reported, but some studies suggest overall positive effects on milk performance, especially under heat stress [12,14]. Conversely, several studies have demonstrated that supplementing dairy cows with GABA, particularly in rumen-protected form, prevents a decrease in milk production and milk protein content, especially during periods of heat stress [12,13]. On the other hand, a previous study reported a quadratic response to GABA supplementation levels, with milk protein increases being greater at lower doses [12]. These results suggest that milk protein response to GABA is not dose-dependent beyond certain levels. Although there is no established direct biochemical correlation between the activity of GABA and lactose concentrations, GABA participates in insulin and glucose regulation, both of which are essential for lactose synthesis [22]. Although the precise mechanism underlying the GABA supplementation effect in milk fat remains unclear, multiple studies have established that GABA participates in carbohydrate metabolism and contributes to improved energy metabolism in dairy cows. The behavioral changes observed during the experimental period following rumen-protected GABA supplementation clearly demonstrate rumen-protected GABA’s effectiveness. In the rumen-protected GABA treatment group, both activity time and feed intake time increased significantly, while rest and rumination times decreased significantly. Previous studies have consistently confirmed that GABA supplementation leads to increased feed intake time, changes in rumination and rest patterns, and reduced oxidative stress in the body [12,13]. However, the restricted feeding protocol in this study prevented assessment of GABA’s effect on voluntary feed intake, which increased in previous studies.

Overall, supplementation with rumen-protected GABA appears to beneficially reduce heat generated from ruminal fermentation by providing additional nutrients during periods of heat stress. This nutritional strategy increases feed intake and helps prevent productivity losses in ruminants exposed to high ambient temperatures, primarily through positive physiological and metabolic effects.

## 5. Conclusions

This study evaluated the effects of varying levels of rumen-protected GABA supplementation on productivity, blood biochemical parameters, and behavioral changes in Holstein dairy cows exposed to heat stress. Although mean milk yield did not differ significantly across treatments, increased levels of rumen-protected GABA tended to mitigate the decline in milk yield associated with heat stress. This observation aligns with previous findings that GABA supplementation can help alleviate heat stress impacts, partly by improving feed intake and nutrient utilization. Behavioral assessments further revealed longer feed intake time and reduced rest and rumination times in cows receiving higher GABA supplementation, supporting GABA’s role in promoting metabolic adaptation and reducing oxidative stress under thermal challenge. This study suggests that daily supplementation with 3 g of ruminally protected GABA may attenuate productivity decline in dairy cows during heat stress. Consequently, ruminally protected GABA represents a practical nutritional intervention for minimizing productivity losses in Holstein cows during periods of elevated ambient temperature.

## Figures and Tables

**Table 1:** Experiment grouping considering days in milk, milk yield, and parity in early-lactation cows.

	Treatment ^1^		
Item	Control	T-1	T-2
Head, no	6	6	6
BW ^1^, kg	263.5	274.5	298.8
Days in milk, day	86.67	86.17	84.17
Milk yield, kg/d	34.20	34.33	34.32
Parity	2.33	2.17	2.17

^1^ The BW of the experimental animal did not differ among treatments (*p* = 0.2092).

**Table 2:** Formula and chemical composition of experimental total mixed ration diets for dairy cows.

Formula (As-Fed Basis)	Composition (%)
Concentrate feed	38.2
Timothy hay	12.8
Alfalfa hay	7.6
Tall fescue	13.8
Water	27.5
Total	100.0
Chemical composition ^1^	
Dry matter (%)	65.0
Moisture (%)	35.0
Crude protein (%DM)	14.6
Crude fiber (%DM)	24.8
NDF (%DM)	49.8
ADF (%DM)	32.0
Ca (%DM)	0.9
P (%DM)	0.6
TDN ^1^	68.1

^1^ NDF, neutral detergent fiber; ADF, acid detergent fiber; TDN, total digestible nutrients.

**Table 3:** Behavior classification of dairy cows in this study.

Classification	Definition
High Activity	Refers to periods when cows exhibit vigorous physical movement. Examples include energetic walking, running, mounting, or other actions that result in high levels of body acceleration. High activity is often associated with excitement, herd movement, estrus behavior, or response to environmental changes.
Activity	Denotes routine movement and normal posture changes such as walking, standing, shifting position at the feed bunk, or exploring their environment. This level of activity is considered moderate and is a sign of a healthy, engaged cow.
Rest	Refers to periods when a cow is lying down or standing still with minimal movement. Rest is essential for rumination, milk production, and overall health. Cows typically rest lying down for several hours a day, and insufficient rest can be a marker for stress or health issues.
Eat (Feeding)	Indicates when cows are actively ingesting feed. This is characterized by a head-down posture at the manger, feed intake, and rhythmic jaw movements as they chew. Eating is distinct from rumination and can be measured by time spent at the feed bunk or feeding station.
Rumination	Refers to the process of chewing cud, where cows regurgitate previously swallowed feed and chew it again to aid digestion. Rumination is marked by slow, methodical jaw movements; it usually occurs when cows are resting (lying or standing) and is crucial for efficient digestion and milk production. Abnormal rumination times can indicate health problems.

**Table 4:** Ambient temperature, relative humidity, and temperature–humidity index during the experimental period.

Items	Experimental Period ^1^, Week								
	0	1	2	3	4	5	6	7	8
AT, °C	27.5	27.7	28.8	29.5	29.3	29.2	27.0	26.7	26.7
RH, %	82.8	85.9	84.1	82.9	80.9	79.2	99.9	76.2	80.5
THI ^2^									
Min	74.7	76.0	78.2	78.4	77.2	76.8	73.2	69.6	70.5
Mean	79.1	79.7	81.9	82.9	81.8	81.5	79.2	75.6	75.7
Median	79.1	79.7	81.9	82.3	81.3	81.0	78.7	74.8	75.4
Max	85.0	83.7	86.4	88.0	87.1	86.8	85.7	81.8	81.0
THI classification, % of day									
under 72	0.0	0.0	0.0	0.0	0.0	0.0	8.3	23.5	18.1
73 to 80	64.2	55.6	32.5	22.6	34.5	36.1	45.4	58.5	65.6
81 to 89	35.8	44.4	67.5	77.4	65.5	63.9	44.3	18.0	16.3
over 90	0.0	0.0	0.0	0.0	0.0	0.0	2.0	0.0	0.0
THI_ratio ^3^	−135.8	−144.4	−167.5	−177.4	−165.5	−163.9	−131.8	−71.0	−80.1

AT, ambient temperature; RH, relative humidity; THI, temperature–humidity index. ^1^ 0 week, 17 July 2024; 8 week, 2024.09.11. ^2^ THI = (1.8 × ambient temperature + 32) − (0.55 − 0.0055 × relative humidity) × (1.8 × ambient temperature − 26) [7]. ^3^ THI ratio = 1 × Daily percentage under THI 72 − 1 × Daily percentage under THI 72 to 78—2 × Daily percentage under THI 78 to 89 − 3 × Daily percentage over THI 90 [15].

**Table 5:** Effects of rumen-protected GABA on milk yield and rectal temperature during heat stress (HS) in lactating Holstein cows.

	Treatment ^1^				*p*-Value ^2^		
Item	Con	T-1	T-2	SEM	GABA	Week	GABA × Week
Milk production ^3^							
Milk yield, kg/d	33.0	34.2	35.5	0.79	0.083	0.676	1.000
3.5% FCM	33.8	35.3	36.8	0.97	0.088	0.001	0.975
FPCM	31.8	32.9	34.2	0.88	0.166	0.001	0.982
RT, °C	38.1	38.3	38.3	0.09	0.341	0.001	0.786

SEM, standard error of the means; 3.5% FCM, fat-corrected milk; FPCM, fat–protein-corrected milk; RT, rectal temperature. ^1^ Control, no additives; T-1, rumen-protected GABA 3 g/head/day; T-2, rumen-protected GABA 6 g/head/day. ^2^ GABA, effect of dietary GABA supplementation; Week, effect of experimental time; GABA × Week, interaction between control and dietary GABA supplementation effect. ^3^ Daily milk yield was calculated as the sum of morning and afternoon milking values.

**Table 6:** Effects of rumen-protected GABA on milk compositions under heat stress in lactating Holstein cows.

	Treatment ^1^				*p*-Value ^2^		
Item	Con	T-1	T-2	SEM	GABA	Week	GABA × Week
Fat, %	3.42 ^b^	3.80 ^a^	3.78 ^a^	0.12	0.036	0.001	0.614
Protein, %	3.06	3.05	2.98	0.02	0.055	0.001	1.000
Lactose, %	4.81 ^b^	4.83 ^ab^	4.88 ^a^	0.02	0.017	0.030	0.794
Solids-not-fat, %	8.52	8.54	8.51	0.04	0.894	0.001	0.999
Milk urea nitrogen, mg/dL	13.6	13.5	12.9	0.27	0.181	0.001	0.683
Somatic cell count, cells/L	0.230	0.193	0.262	0.031	0.273	0.961	0.856

SEM, standard error of the means; GABA, gamma-aminobutyric acid. ^1^ Control, no additives; T-1, rumen-protected GABA 3 g/head/day; T-2, rumen-protected GABA 6 g/head/day. ^2^ GABA, effect of dietary GABA supplementation; Week, effect of experimental time; GABA × Week, interaction between control and dietary GABA supplementation effect. ^ab^ Mean significantly differ in row among treatments (*p* < 0.05).

**Table 7:** Effects of rumen-protected GABA on metabolic profiles and cortisol during heat stress in lactating Holstein cows.

	Treatment ^1^				*p*-Value ^2^		
Item	Con	T-1	T-2	SEM	GABA	Week	GABA × Week
Glucose, mg/dL	37.8	39.7	39.7	1.38	0.539	0.001	0.932
Albumin, g/dL	3.63	3.73	3.70	0.05	0.386	0.233	0.814
Total cholesterol, mg/dL	169.8	186.4	180.9	10.57	0.530	0.010	0.994
NEFA, uEq/L	174.3	151.8	147.4	25.14	0.722	0.075	0.934
BUN, mg/dL	17.4	16.7	16.6	0.54	0.474	0.043	0.676
TP, g/dL	7.75	7.63	7.54	0.09	0.288	0.002	0.936
AST, U/L	132.4	126.9	127.0	12.03	0.933	0.279	0.939
ALT, U/L	28.1	28.3	27.7	1.40	0.944	0.053	0.986
Ca, mg/dL	9.67	9.88	9.97	0.11	0.130	0.000	0.410
P, mg/dL	5.88	6.62	6.18	0.25	0.128	0.007	0.939
Mg, mg/dL	2.27	2.39	2.33	0.08	0.533	0.876	0.526
Cortisol, ug/dL	0.66	0.66	0.73	0.12	0.903	0.229	0.715

SEM, standard error of the means; GABA, gamma-aminobutyric acid; NEFA, non-esterified fatty acid; BUN, blood urea nitrogen; TP, total protein; AST, aspartate aminotransferase; ALT, alanine aminotransferase. ^1^ Control, no additives; T-1, rumen-protected GABA 3 g/head/day; T-2, rumen-protected GABA 6 g/head/day. ^2^ GABA, effect of dietary GABA supplementation; Week, effect of experimental time; GABA × Week, interaction between control and dietary GABA supplementation effect.

**Table 8:** Effects of rumen-protected GABA on behaviors during heat stress in lactating Holstein cows.

	Treatment ^1^				*p*-Value ^2^		
Behavior, % of day	Con	T-1	T-2	SEM ^3^	GABA	Week	GABA × Week
High activity	6.57	8.16	8.38	0.271	0.001	0.001	0.998
Activity	16.88	18.61	18.88	0.535	0.017	0.001	0.998
Rest	23.15	20.75	19.73	0.987	0.043	0.001	1.000
Eat	12.44	13.25	14.52	0.607	0.051	0.001	1.000
Rumination	41.07	39.00	38.33	0.541	0.001	0.001	0.994

^1^ Control, no additives; T-1, rumen-protected GABA 3 g/head/day; T-2, rumen-protected GABA 6 g/head/day. ^2^ GABA, effect of dietary GABA supplementation; Week, effect of experimental time; GABA × Week, interaction between control and dietary GABA supplementation effect. ^3^ SEM, standard error of the means.

## Data Availability

Data are contained within the article.
